# Supplementary figures and images for: Identification of plasma proteins associated with seizures in epilepsy: A consensus machine learning approach
Source: PLoS One. 2025 Jul 1;20(7):e0327317. doi: 10.1371/journal.pone.0327317 (PMC12212537; doi:10.1371/journal.pone.0327317)

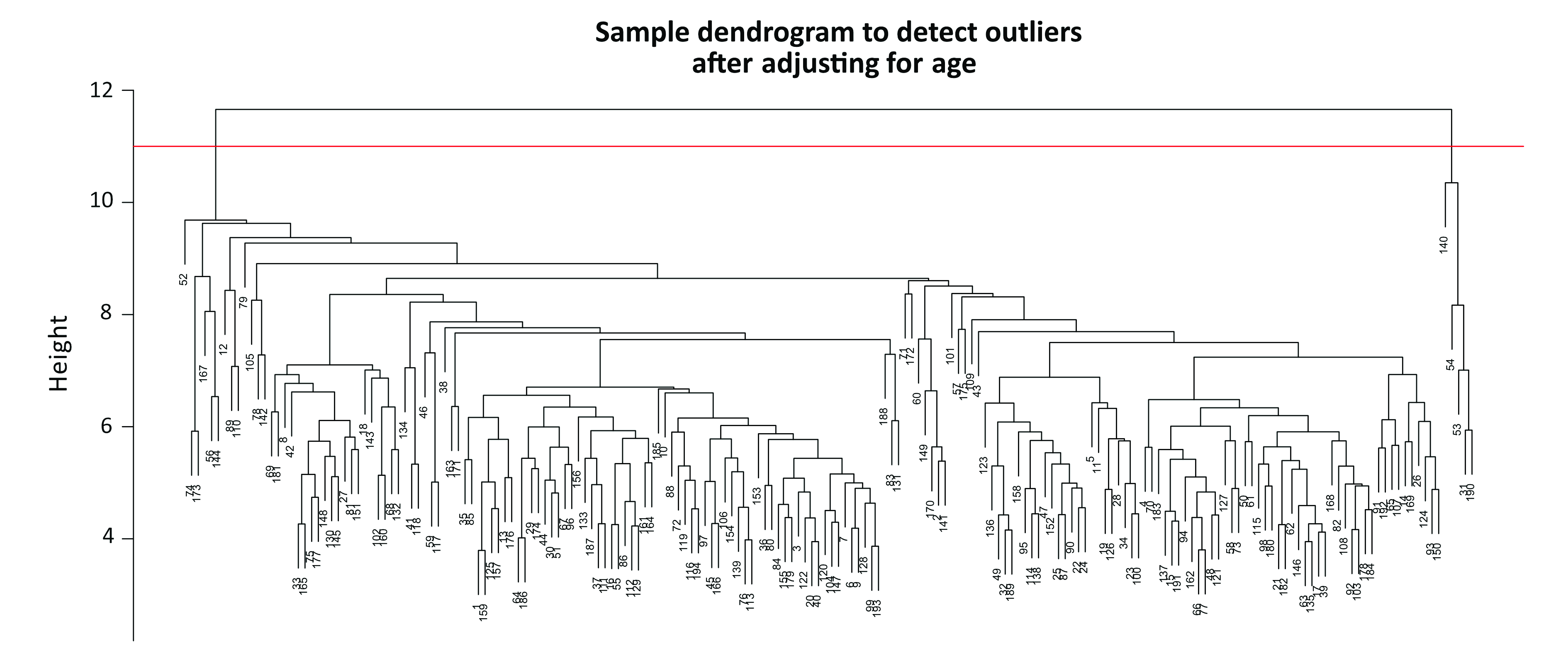

Supplement: S1 Fig — The distance measure was “Euclidean distance” for calculating the distance matrix. The agglomeration method for the hierarchical clustering function was “Average”. The clustering dendrogram shows that the outliers are separate from the main cluster at a height of greater than 10. (TIF) [file pone.0327317.s001.tif]

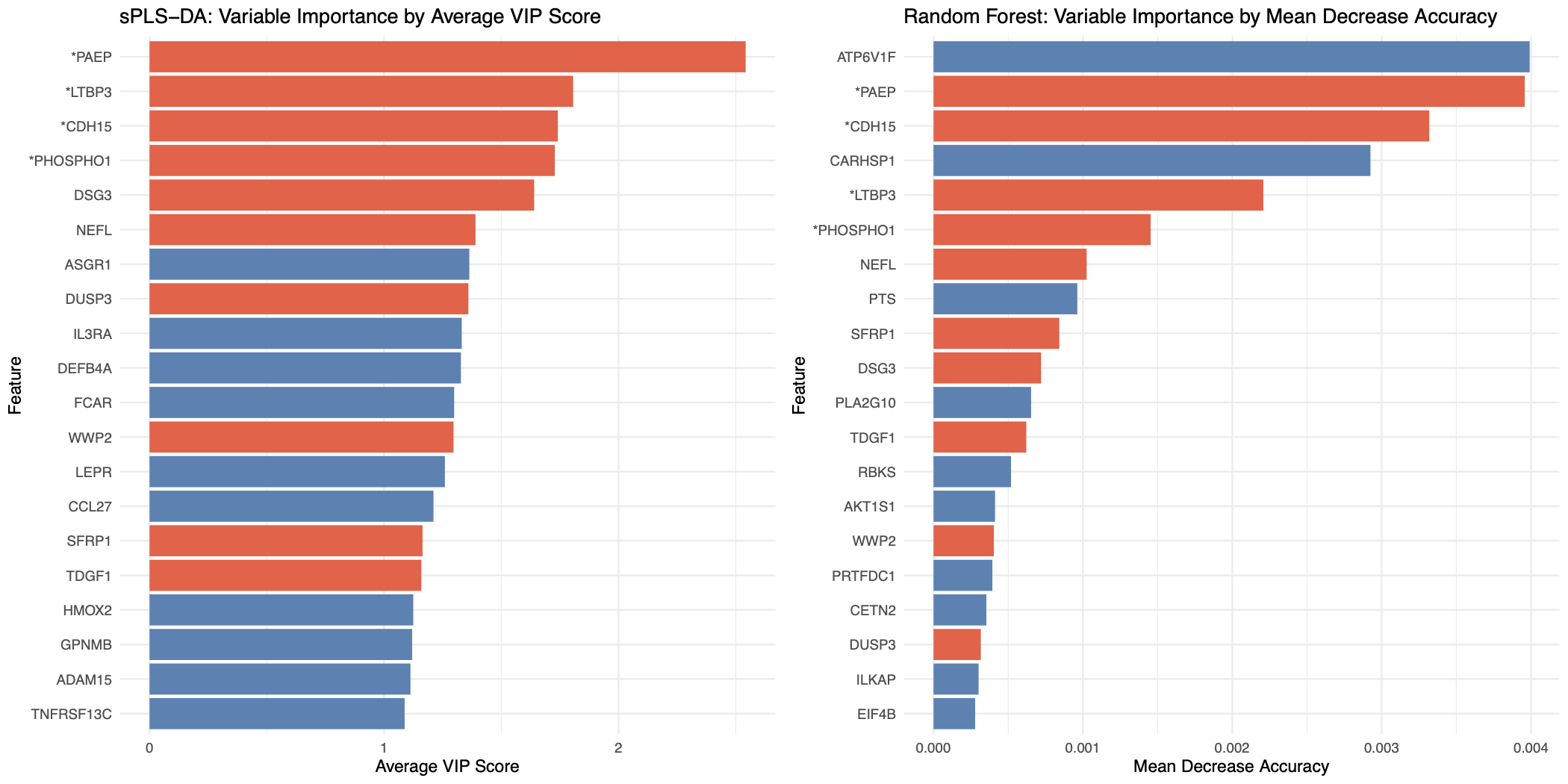

Supplement: S2 Fig — After training the sPLS-DA model, we chose the top explanatory variables (proteins) with variable importance (VIP) > 1. Then to keep the balance between linearity and non-linearity of associations with the seizure status, we chose the same number of top VIP-ranked explanatory variables (proteins) from the trained Random Forest model (20 proteins). Finally, we chose the intersection of the mentioned top explanatory variables (and called them consensus proteins) which are shown to be important (using the ranked VIP) in discriminating between seizure and seizure-free states. (TIF) [file pone.0327317.s002.tif]
