# Supplementary material for: Identification of plasma proteins associated with seizures in epilepsy: A consensus machine learning approach
Source: PLoS One. 2025 Jul 1;20(7):e0327317. doi: 10.1371/journal.pone.0327317 (PMC12212537; doi:10.1371/journal.pone.0327317)
Supplement: Attachment — Submitted filename: response_to_reviewers_PLOS_ONE_20250522.docx [file pone.0327317.s007.docx]

Manuscript Number: **PONE-D-24-55733**

Dear Dr. Ginsberg,

Thank you for the decision letter and the reviewer comments. We have made multiple clarifications and added new analyses as suggested, which has in our opinion substantially improved the paper. Specifically, we now explain the rationale for the selected markers and have added a new AUC analysis showing how much discriminative value they add in comparison to the markers previously identified in traditional analyses. The new analysis originates in a most useful reviewer suggestion; and illustrates both how the machine learning-approach found additional proteins beyond the standard analyses and that single marker analysis may not suffice in future proteomics studies in epilepsy. We have addressed all reviewer comments and the comments from the editorial office. We have also altered the abstract to reflect the new results.

For the authors,

Saman Hosseini Ashtiani, Ph.D.

Specifically, we have made the following changes:

## Reviewer #1:

The article is original and very relevant for the field. This is a comprehensive metabolomics approach which analyzed OLINK proteomics data from a large epilepsy cohort in which they have previously found four differentially expressed proteins (CDH15, PAEP, LTBP3, PHOSPHO1) in patients with recent seizures. Using two machine-learning techniques, they confirmed the four previously detected proteins, but additionally uncovered ten consensus candidate protein biomarkers (CDH15, PAEP, LTBP3, PHOSPHO1, NEFL, SFRP1, TDGF1, DUSP3, WWP2 and DSG3) that contributed to classification of patients as being seizure-free or not. The methodology of the study is modern and very complex, using very modern tools for biochemistry analysis and molecular biology. The results conducted to the new findings showing that: using a combination of the four differentially expressed proteins (CDH15, PAEP, LTBP3, PHOSPHO1) as well as the additional consensus proteins (SFRP1, DSG3, DUSP3 and WWP2) not present among their previous findings out performed individual proteins in identifying individuals with recent seizures, highlighting the potential of multi-protein profiles.

The conclusions are consistent with the evidence and arguments presented.

The references are appropriate, including some very relevant authors experience in the field. I recommend some corrections.

**Comment 1:-** Number of lines would be very useful for small pointed corrections.

**Response: Thank you for the encouraging evaluation. Number of lines have been added throughout the manuscript.**

**Comment 2:** Both Introduction and Discussions should be more detailed to include the other biomarkers for epilepsy. For Introduction you may see also M. Musteata et al., 2013, The 1H NMR Profile of Healthy Dog Cerebrospinal Fluid, PLoS ONE 8(12): e81192. doi:10.1371/journal.pone.0081192 M. Armaşu, R.M.A. Packer, S. Cook, G. Solcan, H.A. Volk, An exploratory study using a statistical approach as a platform for clinical reasoning in canine epilepsy, The Veterinary journal, 2014, 202, 292-296, DOI: 10.1016/j.tvjl.2014.08.008

**Response:**

**We have added more detail to the discussion:**

- **Page 10, 512-514 and 526-528**
- **Page 11, 573-575**

**We added the latter of the suggested references to the discussion (page 11, 575).**

## Reviewer #2:

This study used machine learning (Random Forest and sPLS-DA) to analyze plasma proteomics data from individuals with epilepsy, aiming to identify biomarkers linked to recent seizures. Ten candidate proteins were identified, including four previously known and six novel markers. The authors suggest that combination of proteins improved seizure classification accuracy, highlighting the potential for blood-based biomarkers in epilepsy monitoring. The study is relevant as there is an unmet need for biomarkers however there are certain aspects of the work that need improvement to increase the strength of the manuscript and comprehension of the work for the reader.

Comments:

**Comment 1:*** Review manuscript for grammatical or typographical errors. Key sentences in the manuscript need to be reviewed to be written in a clearer and more concise way.

-The title of the manuscript has typos.

-This sentence has grammar errors -” Using the four candidate markers together corroborate the utility of proteomic profiling in epilepsy research and highlight the importance of studying profiles of marker expression in addition to individual proteins. “

- Page 9, last sentence has a typo.

- This sentence has grammar errors - “Some patients with epilepsy refractory to traditional epilepsy medications respond to immunotherapy, suggesting that a better understanding of immune pathways on an individual level are likely to be integral to epilepsy care in the future.”

-Key sentence to be written clearly –“ Four proteins (PAEP, CDH15, LTBP3 and PHOSPHO1) were demonstrated to have significant association with the seizure status (p-value < 0.05) using both sPLS-DA and Random Forest models besides the rest of the proteins used for classification in both classifiers that were not among our findings in the previous study (SFRP1, DSG3, DUSP3 and WWP2).”

**Response:**

**The grammatical and typo errors have been fixed including:**

- **The title’s typo has been fixed**
- **Page 11, 572-574**
- **Page 9, 413-414**
- **Page 10, 537**
- **Pages 5 and 6, 247-292**

**Comment 2:*** Stronger reasoning is necessary for the selection of the 4 novel proteins (SFRP1, DUSP3, WWP2 and DSG3 ) since they were not significantly differentially expressed in any of the methods - sPLS-DA and Random Forest.

**Response:**

**We have now clarified this very important point:**

- **Pages 5, 215-228**
- **Pages 5 and 6, 247-292**
- **Pages 8 and 9, 366-384**

**Comment 3:*** In the discussion the authors bring up “FCAR” related to inflammation, but it has not been mentioned or discussed in the data before. “FCAR (Fc Alpha Receptor) Binds to the Fc region of immunoglobulins alpha and mediates several functions including cytokine, superoxide radicals and inflammatory radicals.” production.

**Response:**

**This was a residue from a previous manuscript version, and has been removed.**

**Comment 4:*** More information is needed in the figure descriptions and tables to include statistical tests performed for the analysis. Additionally, the AUC in the description of Figure 2 do not match with the AUC in the figure. Label correctly the figures when attaching.

**Response:**

**The figure captions and table descriptions have been corrected and/or improved:**

- **Figure 1 caption: Page 6, 294-299**
- **Figure 2 caption: Page 6, 301-304**
- **Table 2 caption: Page 7, 342-345**
- **Table 3 caption: Page 8, 353-355**
- **Figure 3 caption: Page 9, 386-392**
- **Figure 4 caption: Page 9, 395-404**
- **Supporting information captions: 754-778**

**Comment 5:*** In the discussion of Figure 3, the authors mention that the combination of four proteins outperformed any of these previously identified proteins when used individually to predict individuals with recent seizures. However, in Figure 3B, DUSP3 has an AUC=1 suggesting that the protein perfectly distinguishes between seizure and seizure-free individuals without any error. This is either a typo or an issue in the analysis.

**Response:**

**This was a plotting error, which has been fixed (Figure 3)**

6. PLOS authors have the option to publish the peer review history of their article (what does this mean?). If published, this will include your full peer review and any attached files.

If you choose “no”, your identity will remain anonymous but your review may still be made public.

Do you want your identity to be public for this peer review? For information about this choice, including consent withdrawal, please see our Privacy Policy.

## Reviewer #1: Yes: G. Solcan

## Reviewer #2: No

**Response:**

**Yes, we have no objection to the reviews being published. It shows very nicely how the additional analysis of all biomarkers added to the manuscript.**

**We have also addressed the following editorial comments.**

1.Please ensure that your manuscript meets PLOS ONE's style requirements, including those for file naming. The PLOS ONE style templates can be found at

<https://journals.plos.org/plosone/s/file?id=wjVg/PLOSOne_formatting_sample_main_body.pdf> and

[https://journals.plos.org/plosone/s/file?id=ba62/PLOSOne_formatting_sample_title_authors_affiliations.pdf](https://track.editorialmanager.com/CL0/https:%2F%2Fjournals.plos.org%2Fplosone%2Fs%2Ffile%3Fid=ba62%2FPLOSOne_formatting_sample_title_authors_affiliations.pdf/1/010f0195ccbe3a63-981b1f31-109a-461d-b9e2-77cdc8490fde-000000/xSRnDOIdmdI1b06PGY8wj2exkhDqfJ_6RCV0r-Gd_Ms=203)

**Response:**

**We have updated the format and styles of the manuscript.**

2. Please note that PLOS ONE has specific guidelines on code sharing for submissions in which author-generated code underpins the findings in the manuscript. In these cases, we expect all author-generated code to be made available without restrictions upon publication of the work. Please review our guidelines at <https://journals.plos.org/plosone/s/materials-and-software-sharing#loc-sharing-code> and ensure that your code is shared in a way that follows best practice and facilitates reproducibility and reuse.

**Response:**

**All the codes will be publicly available via GitHub repository after the probable acceptance.**

3. We note that the grant information you provided in the ‘Funding Information’ and ‘Financial Disclosure’ sections do not match.

When you resubmit, please ensure that you provide the correct grant numbers for the awards you received for your study in the ‘Funding Information’ section.

**Response:**

**We have made sure that the grant numbers are correct and consistent.**

4. Thank you for stating the following financial disclosure:

“This study was funded by grants from Jeansson foundation, Swedish Society for Medical Research (SS18-0040), Region Västra Götaland (VGFOUREG-968476), Swedish state under the agreement between the Swedish government and the county councils and the ALF-agreement (715781).”

Please state what role the funders took in the study.  If the funders had no role, please state: "The funders had no role in study design, data collection and analysis, decision to publish, or preparation of the manuscript."

If this statement is not correct you must amend it as needed.

Please include this amended Role of Funder statement in your cover letter; we will change the online submission form on your behalf.

**Response:**

**The amended statement has been added within the cover letter.**

5. Thank you for stating the following in the Competing Interests section:

“J.Z. received speaker honoraria from UCB and Eisai for non-branded education events; and as employee of Sahlgrenska University Hospital is or has been an investigator/sub investigator in clinical trials sponsored by GW Pharma, SK life science, UCB, Angilini Pharma, and Bial (no personal compensation).”

We note that you received funding from a commercial source: UCB and Eisai

Please provide an amended Competing Interests Statement that explicitly states this commercial funder, along with any other relevant declarations relating to employment, consultancy, patents, products in development, marketed products, etc.

Within this Competing Interests Statement, please confirm that this does not alter your adherence to all PLOS ONE policies on sharing data and materials by including the following statement: "This does not alter our adherence to PLOS ONE policies on sharing data and materials.” (as detailed online in our guide for authors <http://journals.plos.org/plosone/s/competing-interests>).  If there are restrictions on sharing of data and/or materials, please state these. Please note that we cannot proceed with consideration of your article until this information has been declared.

Please include your amended Competing Interests Statement within your cover letter. We will change the online submission form on your behalf.

**Response:**

**The amended statement has been added within the cover letter.**

6. We note that you have indicated that there are restrictions to data sharing for this study. PLOS only allows data to be available upon request if there are legal or ethical restrictions on sharing data publicly. For more information on unacceptable data access restrictions, please see <http://journals.plos.org/plosone/s/data-availability#loc-unacceptable-data-access-restrictions>.

Before we proceed with your manuscript, please address the following prompts:

a) If there are ethical or legal restrictions on sharing a de-identified data set, please explain them in detail (e.g., data contain potentially identifying or sensitive patient information, data are owned by a third-party organization, etc.) and who has imposed them (e.g., a Research Ethics Committee or Institutional Review Board, etc.). Please also provide contact information for a data access committee, ethics committee, or other institutional body to which data requests may be sent.

b) If there are no restrictions, please upload the minimal anonymized data set necessary to replicate your study findings to a stable, public repository and provide us with the relevant URLs, DOIs, or accession numbers. For a list of recommended repositories, please see

<https://journals.plos.org/plosone/s/recommended-repositories>. You also have the option of uploading the data as Supporting Information files, but we would recommend depositing data directly to a data repository if possible.

We will update your Data Availability statement on your behalf to reflect the information you provide.

**Response:**

**Data Availability Statement has been added to page 15: 783-791.**

**The underlying data in this study are sensitive personal data according to the Swedish Data Protection Regulation. The ethical approval of the Swedish Ethical Review Authority for this study (2020-853) states that the data and results can only be published on a group level, i.e., without any patient-level details. Both original and translated ethical approvals are uploaded. The contact details for the Swedish Ethical Review Authority that has imposed such restrictions along with a link to clear explanations identifying any patient-level health-related data as sensitive:**

[**registrator@etikprovning.se**](mailto:registrator@etikprovning.se)

**Phone: +46104750800**

[**https://www.imy.se/en/organisations/data-protection/data-protection-within-different-areas/processing-of-personal-data--for-researchers/**](https://www.imy.se/en/organisations/data-protection/data-protection-within-different-areas/processing-of-personal-data--for-researchers/)

**Post address:**

**Etikprövningsmyndigheten**

**Box 2110**

**750 02 Uppsala**

7. Please include your full ethics statement in the ‘Methods’ section of your manuscript file. In your statement, please include the full name of the IRB or ethics committee who approved or waived your study, as well as whether or not you obtained informed written or verbal consent. If consent was waived for your study, please include this information in your statement as well.

**Response:**

**The information has been added to lines 157-159 of the “Methods” section.**
